# Performance of Fully Automated Antimicrobial Disk Diffusion Susceptibility Testing Using Copan WASP Colibri Coupled to the Radian In-Line Carousel and Expert System

**DOI:** 10.1128/JCM.00777-21

**Published:** 2021-08-18

**Authors:** Abdessalam Cherkaoui, Gesuele Renzi, Nicolas Vuilleumier, Jacques Schrenzel

**Affiliations:** a Bacteriology Laboratory, Division of Laboratory Medicine, Department of Diagnostics, Geneva University Hospitals, Geneva, Switzerland; b Division of Laboratory Medicine, Department of Diagnostics, Geneva University Hospitals and Faculty of Medicine, Geneva, Switzerland; c Genomic Research Laboratory, Division of Infectious Diseases, Department of Medicine, Geneva University Hospitals and Faculty of Medicine, Geneva, Switzerland; NorthShore University HealthSystem

**Keywords:** automation, disk diffusion, antimicrobial susceptibility testing, Copan WASPLab, Colibri, Radian, Vitek 2

## Abstract

The purpose of the present study was to assess the agreement at the categorical level between the Vitek 2 system and the Colibri coupled to the Radian under real routine laboratory conditions. The 675 nonduplicate clinical strains included in this study (249 *Enterobacterales* isolates, 198 Pseudomonas aeruginosa, 107 Staphylococcus aureus, 78 coagulase-negative staphylococci, 38 Enterococcus faecalis, and 5 Enterococcus faecium) were isolated from nonconsecutive clinical samples referred to our laboratory between June and November 2020. In addition, 43 carbapenemase-producing *Enterobacterales* (CPE) formerly identified and stored in our laboratory were added to the panel, for a total of 718 strains. The overall categorical agreements between the two compared methods were 99.3% (4,350/4,380; 95% CI 99% to 99.5%); 98.6% (2,147/2,178; 95% CI 98.0% to 99.0%); 99.4% (1,839/1,850; 95% CI 98.9% to 99.7%); and 99.4% (342/344; 95% CI 97.9% to 99.8%) for *Enterobacterales*, P. aeruginosa, Staphylococcus spp., and *Enterococcus* spp., respectively. The most important cause of the very major errors encountered on the Vitek 2 for P. aeruginosa (62%, 13/21) was related to the presence of heteroresistant populations. Among the 43 CPE included in this study, one OXA-48-like, and one OXA-181-like were missed by the Vitek 2, even by rigorously applying the CPE screening cutoffs defined by EUCAST. The Colibri coupled to the Radian provide a fully automated solution for antimicrobial disk diffusion susceptibility testing with an accuracy that is equal to or better than that of the Vitek 2 system.

## INTRODUCTION

One of the most important tasks of clinical bacteriology laboratories is to perform accurate antimicrobial susceptibility testing (AST) on the relevant clinical bacterial isolates. The main objective of AST consists of indicating the best antimicrobial molecules for treating the tested organism. Currently, the list of bacterial pathogens for which narrow-spectrum empirical therapy remains effective has become increasingly shorter, making AST necessary to rapidly deescalate and target antimicrobial therapy. Since the first publications on antibiotic susceptibility testing in 1954 (1), a large number of assays has been made available to assist laboratories and clinicians in selecting the appropriate antimicrobial therapy. Most commonly used testing methods encompass rapid semiautomated commercial instruments, broth microdilution, Kirby-Bauer disk diffusion, and gradient diffusion. Among them, the latter two methods provide the most flexibility. Over the years, users have reported the strengths and weaknesses of each method, including the list of bacteria that can be accurately tested with the ability to detect different antimicrobial resistance mechanisms (2–4). The testing methods either return quantitative results expressed in MIC values or provide only qualitative results (susceptible or resistant). Basically, current testing methods detect accurately the most common antimicrobial resistance mechanisms; in that sense, emerging or recently reported mechanisms warrant constant vigilance to make sure they are correctly detected. The greatest advantage of semiautomated instrument systems compared to manual methods is that the instrumentation enables standardized reading of the end points and swift result returns because the optical systems detect tenuous changes in bacterial growth. Among the four semiautomated instruments validated by the FDA, the Vitek 2 (bioMérieux), based on repetitive turbidimetric monitoring of bacterial growth during a short incubation period, is widely used in clinical microbiology laboratories around the world. The last two decades have heralded a trend away from the Kirby-Bauer disk diffusion in favor of the semiautomated testing systems. There are several reasons for this trend: (i) the accuracy of disk diffusion susceptibility testing relies on high-quality reagents (antibiotic disks and culture media), skilled laboratory procedures, and correct handling of materials; and (ii) automation is only partial; several steps remain manual (e.g, media plating, distribution of antibiotic disks, reading and interpreting the inhibition zones, typing results in the laboratory information system). Overall, the disk diffusion method is time consuming and its interpretation is more error-prone. However, the advent of full automation in microbiology laboratories should enable disk diffusion testing to become one of the major methods to deal with the emergence of new resistance mechanisms while accommodating the ever-increasing activity with a minimal workload and a high traceability.

To assess the performances of the fully automated antimicrobial disk diffusion susceptibility testing provided by Copan WASP Srl (Brescia, Italy), which consists of the Colibri, the Radian in-Line Carousel, and the Radian Expert System, it is necessary to demonstrate that it provides equal or better accuracy than commonly used AST methods and that it can be applied to a broad diversity of clinically relevant microorganisms. The overarching objective of this study was to assess the agreement at the categorical level between the Vitek 2 system and the Colibri coupled to the Radian for AST under real routine laboratory conditions. Outcome measures included the accuracy of identification to the species level, throughput, and workflow.

## MATERIALS AND METHODS

### Setting.

This study was performed in the bacteriology laboratory of Geneva University Hospitals, a Swiss tertiary care center with more than 1,900 beds. The normal hours of operation extend from 07:30 a.m. to 10:00 pm (7/7). About 165,000 clinical samples and 25,000 AST panels are processed annually. All AST panels are performed on the Vitek 2 system or the SIRscan (approximately 50:50).

### Bacterial strains.

The clinical strains included in this study consisted of 675 nonduplicate strains (249 *Enterobacterales* isolates, 198 Pseudomonas aeruginosa, 107 Staphylococcus aureus, 78 coagulase-negative staphylococci, 38 Enterococcus faecalis, and 5 Enterococcus faecium) isolated from nonconsecutive clinical samples referred to our bacteriology laboratory between June and November 2020. In addition, 43 carbapenemase-producing *Enterobacterales* (CPE), formerly identified and stored at −80°C in skim milk with 15% glycerol, were included in the panel, for a total of 718 clinical strains. All stored strains were passaged twice before testing. Table 1 depicts the *Enterobacterales* species and CPE included in this study. The identification of the strains was performed by matrix-assisted laser desorption ionization–time of flight mass spectrometry (MALDI-TOF MS) (Bruker Daltonics, Bremen, Germany) according to the manufacturer's instructions. The confirmation of the extended-spectrum β-lactamase (ESBL) profile was performed by double-disk synergy test (DDST20). For this test, an amoxicillin-clavulanate disk was automatically placed by Radian at 20 mm, center to center, of a cefepime disk on Mueller-Hinton E (MHE) agar, according to a previously reported method (5). In the primary MHE agar plates, the amoxicillin-clavulanate disk was automatically placed by Radian at 27 mm, center to center, of a ceftriaxone disk and a cefepime disk (Fig. S1 and S2 in the supplemental material).

**TABLE 1:** *Enterobacterales* strains included in this study

Species	No. of strains	ESBL^*a*^	NDM^*a*^ producers	KPC^*a*^ producers	OXA-48-like producers	OXA-181-like producers
Escherichia coli	190	16	1	1	4	7
Klebsiella pneumoniae	51	16	3	2	7	6
Klebsiella oxytoca	3	0	0	0	0	0
Klebsiella aerogenes	2	0	0	0	0	0
Proteus mirabilis	20	2	2	0	0	0
Proteus vulgaris	1	0	0	0	0	0
Citrobacter koseri	4	4	0	0	0	4
Citrobacter freundii complex	7	3	0	0	3	0
Serratia marcescens	1	0	0	0	0	0
Enterobacter cloacae complex	7	2	1	0	1	0
Hafnia alvei	2	1	0	0	1	0
Providencia rettgeri	2	0	0	0	0	0
Morganella morganii	2	0	0	0	0	0

Total	292	44	7	3	16	17

a ESBL, extended-spectrum β-lactamases; NDM, New Delhi metallo-β-lactamase; KPC, Klebsiella pneumoniae carbapenemase.

Cefoxitin was tested systematically on all strains of the present study. We also performed the ESBL + AmpC screen kit 98008 (Rosco Diagnostica, Denmark) to identify the partially derepressed AmpC whenever the results of the DDST20 and the cefoxitin were not conclusive (this is especially relevant because cefoxitin has a high sensitivity but poor specificity for identifying the AmpC-producing *Enterobacterales*).

The Eazyplex SuperBug CRE system (Amplex Biosystems GmbH, Giessen, Germany) was used to identify the various CPE. The presence of methicillin-resistant Staphylococcus aureus (MRSA) was confirmed by a previously published quantitative PCR (qPCR) assay targeting *femA* and *mecA* (6).

### Full automation of AST by disk diffusion (Colibri, Radian in-line carousel, and Radian Expert System).

**FIG 1:**
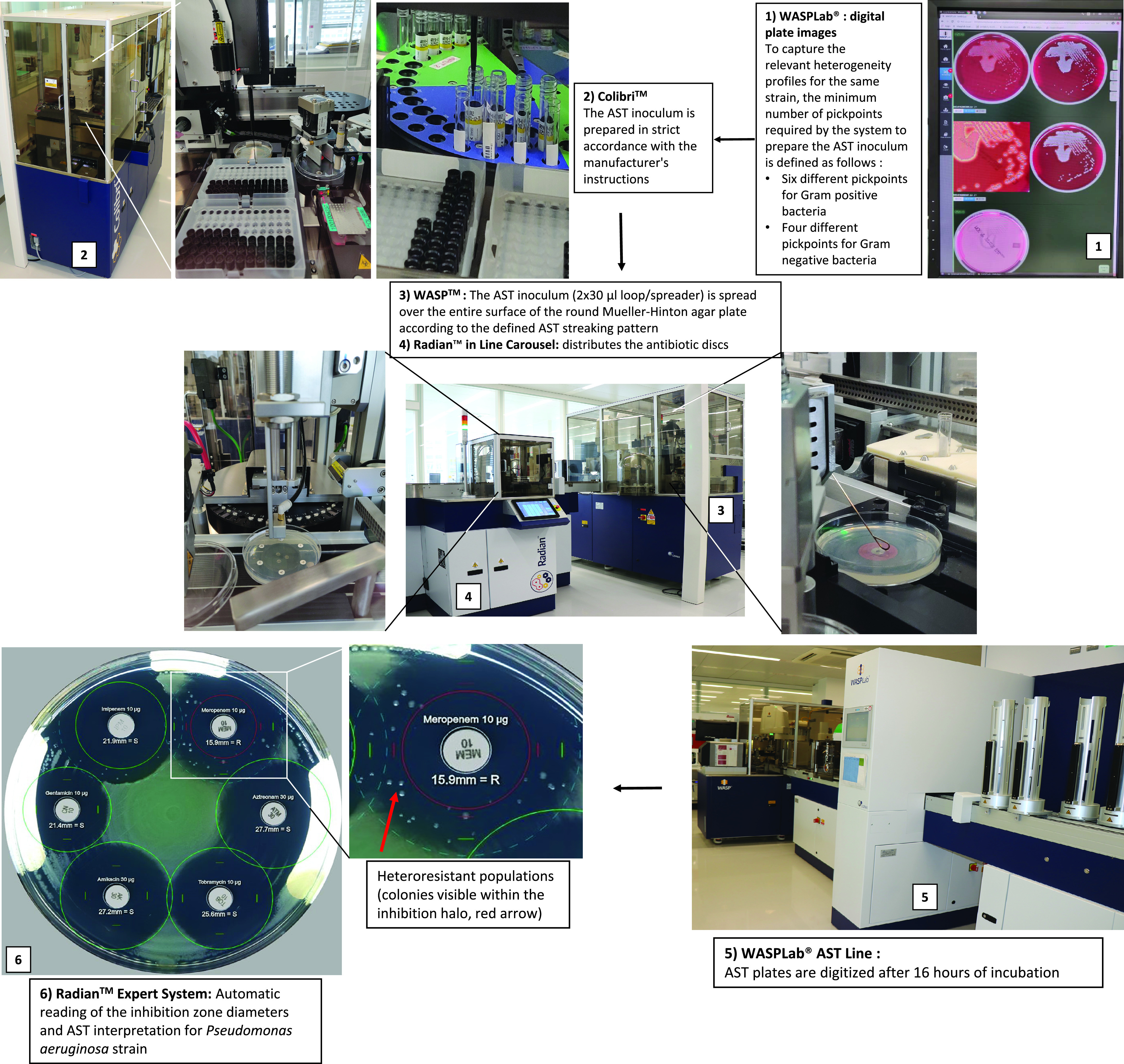
Workflow of a fully automated solution for antimicrobial disk diffusion susceptibility testing: (1) WASPLab digital plate images; (2) Colibri; (3) WASP; (4) Radian in-line carousel; (5) WASPLab AST line; (6) Radian Expert System. Colibri prepares the inocula for 10 strains within 21 min. The AST line (WASP + Radian in-line carousel) executes AST for 10 strains (i.e. 40 culture medium plates and 200 antibiotic discs) within 44 min.

The Radian is a WASPLab module developed by Copan WASP Srl (Brescia, Italy). It is devoted to automating AST by disk diffusion and consists of two units. (i) The Radian in-line carousel, which handles the media plates and dispenses the antibiotic disks. The carousel can contain up to 50 different antibiotic cartridges and enables 1 to 8 antibiotic disk deposit protocols, thereby allowing for automated setup of the double-disk synergy test (DDST 20 mm) (Fig. S1 and S2). (ii) The Radian Expert System is a stand-alone software that is connected to the WASPLab WebApp. This software allows automatic reading of inhibition zone diameters and the interpretation using EUCAST or CLSI rules. The inoculum suspension was prepared by the Colibri in strict accordance with the manufacturer's instruction. The Colibri prepared the inocula of 10 strains within 21 min. Then, that inoculum (2 × 30 µl loop/spreader) was streaked by the WASP over the entire surface of round Mueller-Hinton E (MHE) agar plates (bioMérieux, Geneva, Switzerland) according to the AST streaking pattern defined by Copan, which was previously tested and validated in our previous report (7). The antibiotic disks are then dispensed by the Radian in-line carousel and the inoculated media are transferred by conveyors to the automated incubators (Fig. 1).

All the antibiotic cartridges are stored at 4°C according to the manufacturer’s instructions. The antibiotic cartridges were installed in the Radian carousel only when performing the AST. WASP plus Radian in-line carousel executed AST for 10 strains (i.e., 40 media plates with 200 distributed antibiotic disks) within 44 min. Plates were incubated for 16 h on the WASPLab, and several high-resolution digital images were acquired under different light and exposure conditions according to the manufacturer's instructions. Inhibition zone diameters were automatically read by the WASPLab. The inhibition zone diameters were adjusted manually when deemed necessary, which represented less than 10% of the tested disks. All digital images and the final AST results were validated on the WASPLab screen by microbiologists and experienced technologists without any automatic release of the AST results by the WASPLab.

The AST interpretation was performed by the Radian Expert System according to the EUCAST breakpoints, version 9.0. In this study, we used the i2a antibiotic disks (i2a, Montpellier, France).

### Vitek 2 susceptibility testing.

The AST on the Vitek 2 system was performed in strict accordance with the manufacturer's instruction, as part of routine procedures in our accredited bacteriology laboratory. The EUCAST breakpoints, version 9.0, were applied by the Vitek 2 system. The inoculum suspension was prepared manually by picking a sufficient number of morphologically similar colonies from overnight growth with a sterile stick and by suspending the colonies in sterile saline (aqueous 0.45% to 0.5% NaCl, pH 4.5 to 7.0) to an appropriate McFarland standard using the DensiCHEK Plus. Purity plate checks were performed for all the analyzed strains to ensure that a pure culture was used. We used the following AST cards: AST-N290, AST-N240, AST-P636, and AST-P655 (bioMérieux, Geneva, Switzerland).

### Internal quality controls.

Eight independent biological replicates of Staphylococcus aureus strain ATCC 29213, Escherichia coli strain ATCC 25922, Pseudomonas aeruginosa strain ATCC 27853, and Enterococcus faecalis strain ATCC 29212 were used as internal quality controls to assess the accuracy, reproducibility, and repeatability of the fully automated AST method and of the Vitek 2 system. We also assessed the stability of the antibiotic disks in the Radian in-line carousel. To that end, we performed AST at different time points (15 min, 2 h, 4 h, 5 h, 6 h, 8 h, 10 h, and 11 h 30 min) corresponding to the time elapsed after loading the antibiotic cartridges on the Radian carousel.

### Discordant results.

The fully automated AST results were compared to the Vitek 2 results, the latter being routinely performed in our laboratory. When both methods agreed, we considered the susceptibility category as correct and no further determination was attempted. When the methods gave discordant testing results, we systematically performed broth microdilution (Thermo Scientific Sensititre MIC plates, USA) to resolve the uncertainty. The discordant results were scored as a “very major error” if reported susceptible by the fully automated AST or the Vitek 2 but resistant by broth microdilution, and as a “major error” if scored resistant by the fully automated AST or the Vitek 2 but deemed susceptible by broth microdilution.

Additionally, when the discordant results were related to antimicrobial heteroresistant populations (colonies visible within the inhibition halo of the disk diffusion), we assessed the resistant subpopulations in heteroresistant strains by Etest strips, since various reports have stressed that heteroresistance is accurately detected by this method (8–12).

### Ethical approval

In accordance with the local ethical committee (Commission cantonale d'éthique de la recherche (CCER), https://www.hug-ge.ch/ethique), routine clinical laboratories of our institution may use biological sample leftovers for method development after irreversible anonymization of data.

## RESULTS

### *Enterobacterales*.

Among the 292 *Enterobacterales* strains tested, 30 discordant results were observed at the categorical level; the overall categorical agreement between the compared methods was 99.3% (4,350/4,380; 95% confidence interval [CI] 99% to 99.5%). No discordant results at the categorical level were observed for ampicillin, cefuroxime, or norfloxacin. A total of 15 major errors were observed on the Copan’s fully automated AST. In contrast, 12 very major errors and 3 major errors were observed on the Vitek 2 (Table 2). Importantly, a strict application of the screening cutoff values for carbapenemase-producing *Enterobacterales* (CPE) according to EUCAST methodology, which consists in the use of meropenem (having the best balance of sensitivity and specificity/screening cutoff of MIC > 0.125 mg/liter or inhibition zone diameter of <28 mm) (13), enabled the detection of all 43 CPE included in this study by the Copan’s fully automated AST. The lowest value of MIC determined by the Vitek 2 for meropenem was ≤0.25 mg/liter, which does not permit using the CPE screening cutoff defined by EUCAST, and therefore limiting the possibility to suspect the presence of CPE. As depicted in Table S1, one strain producing OXA-181-like and another strain with OXA-48-like were missed by the Vitek 2 because their MICs for meropenem and ertapenem were ≤0.25 mg/liter and ≤0.5 mg/liter, respectively. Two other strains producing OXA-181-like had meropenem MICs of 1 and 2 mg/liter. By rigorously applying the EUCAST CPE screening cutoffs, these two strains were therefore suspected and then confirmed as CPE by using molecular assays. Obviously, all these four CPE strains were easily suspected as CPE by the Copan’s fully automated AST because ertapenem was reported as resistant and the meropenem inhibition zone diameter was <28 mm (Table S1).

**TABLE 2:** Prevalence (%) of antibiotic resistance phenotypes in the 718 clinical isolates included in this study and categorical agreement between the compared methods

Antibiotics	Resistance rate % (no. of isolates)	Categorical agreement between the compared methods (%)	Colibri coupled to Radian		VITEK 2system	
			Very major error	Major error	Very major error	Major error
*Enterobacterales* species (*n* = 292)						
Ampicillin	66 (193)	100				
Amoxicillin-clavulanate	37 (108)	99.7		1		
Piperacillin-tazobactam	21 (62)	98.6		2	2	
Cefuroxime	25 (73)	100				
Ceftazidime	22 (63)	99.3		2		
Ceftriaxone	22 (63)	99.3		2		
Cefepime	19 (56)	99		1	3	
Imipenem	6 (18)	98.6		2	1	1
Meropenem	7 (19)	99.7			1	
Ertapenem	17 (49)	97.6		3	4	
Amikacin	7 (19)	99.7			1	
Gentamicin	15 (45)	99.7		1		
Norfloxacin	35 (101)	100				
Ciprofloxacin	29 (85)	99.3				2
Cotrimoxazole	35 (103)	99.7		1		
Pseudomonas aeruginosa (*n* = 198)						
Piperacillin	43 (85)	94	1		11 (incl. 5^*a*^)	
Piperacillin-tazobactam	33 (65)	98.5			1	2
Ceftazidime	28 (56)	99.5		1		
Cefepime	28 (55)	99		1	1^*a*^	
Imipenem	30 (60)	98.5	1		2^*a*^	
Meropenem	27 (53)	98			4^*a*^	
Amikacin	24 (47)	99.5				1
Gentamicin	21 (42)	99				2
Tobramycin	23 (46)	100				
Ciprofloxacin	25 (49)	99.5				1
Levofloxacin	31 (61)	99			2 (incl. 1^*a*^)	
Staphylococcus spp. (*n* = 185 including 107 Staphylococcus aureus and 78 coagulase-negative staphylococci)						
Cefoxitine	32 (60)	100				
Gentamicin	21 (39)	100				
Ciprofloxacin	32 (60)	99.5			1	
Clindamycin	29 (53)	100				
Erythromycin	34 (62)	100				
Fusidic acid	26 (48)	100				
Cotrimoxazole	23 (42)	94.6			10	
Rifampin	3 (6)	100				
Tigecyclin	0	100				
Linezolid	0	100				
*Enterococcus* spp. (*n* = 43 including 38 Enterococcus faecalis and 5 Enterococcus faecium)						
Ampicillin	9 (4)	97.7		1		
Imipenem	9 (4)	97.7			1	
Gentamicin	9^*b*^ (4)	100				
Linezolid	0	100				
Teicoplanin	0	100				
Vancomycin	0	100				
Tigecycline	0	100				
Nitrofurantoin	0^*c*^	100				

a Presence of colonies within the inhibition halo (heteroresistance detected only by disk diffusion).

b High level of gentamicin resistance.

c Only Enterococcus faecalis isolates were included.

### Pseudomonas aeruginosa.

Among the 198 Pseudomonas aeruginosa analyzed, we observed 31 discordant results at the categorical level. The overall categorical agreement between the compared methods was 98.6% (2,147/2,178; 95% CI 98.0% to 99.0%). Two very major errors and two major errors were observed on the Copan’s fully automated AST. Twenty-one very major errors and six major errors were observed on the Vitek 2 (Table 2). Among the 21 very major errors recorded on the Vitek 2, 62% (13/21) were linked to heteroresistance profiles that are typically missed by the Vitek 2. In contrast, colonies inside the inhibition halo of the antibiotic disk diffusion were observed for all such strains, indicating the presence of heteroresistant populations (Fig. 1, bottom left; Fig. S3). The identification of all the colonies visible within the inhibition halo was confirmed by MALDI-TOF MS to exclude any contamination.

### Staphylococcus spp.

No discordant results at the categorical level were observed for cefoxitin, gentamicin, clindamycin, erythromycin, fusidic acid, rifampin, linezolid, or tigecycline. Two major errors were observed on the Copan’s fully automated AST for ciprofloxacin (S. aureus) and cotrimoxazole (S. epidermidis). However, 10 very major errors were identified on the Vitek 2 for cotrimoxazole according to broth microdilution results. These very major errors were reported only for coagulase-negative staphylococci (one Staphylococcus hominis and nine Staphylococcus epidermidis) (Fig. S4). The categorical agreement for S. aureus strains was 99.9% (1,069/1,070; 95% CI 99.5% to 100%). The overall categorical agreement between the two compared methods for all the 185 Staphylococcus spp. strains was 99.4% (1,839/1,850; 95% CI 98.9% to 99.7%).

### *Enterococcus* spp.

No discordant results at the categorical level were observed for gentamicin, linezolid, teicoplanin, vancomycin, nitrofurantoin, or tigecycline. One major error was observed on the Copan’s fully automated AST for ampicillin, and one very major error on the Vitek 2 for imipenem. Hence, the overall categorical agreement between the two compared methods for *Enterococcus* spp. strains was 99.4% (342/344; 95% CI 97.9% to 99.8%).

### Internal quality controls.

Careful attention was paid to assess the stability of the antibiotic disks in the Radian in-line carousel. AST on the Copan’s full automation was carried out at specific time points after antibiotic cartridges were loaded on the Radian carousel. We made sure these time points matched the hours of operation in our laboratory. For the eight independent biological replicates of Staphylococcus aureus ATCC 29213, Escherichia coli ATCC 25922, Pseudomonas aeruginosa ATCC 27853, and Enterococcus faecalis ATCC 29212, the inhibition zone diameters were always in the range defined by EUCAST for all the antibiotic disks and at all the different time points assessed (Fig. S5).

No problems were observed for the internal quality controls on the Vitek 2 system.

## DISCUSSION

For many years, there have been only few improvements in the disk diffusion AST. Even with the advent of semiautomatic readers such as SIRscan 2000 Automatic (i2a, Montpellier, France), Adagio (Bio-Rad, USA), or BIOMIC V3 (Giles Scientific, USA) instruments, the manual setup of the disk diffusion and the availability of several automated liquid-based systems for performing AST (Vitek, Phoenix, MicroScan) have thwarted its large scale-use in clinical microbiology laboratories. However, there has been a resurgence of interest lately in disk diffusion because this method offers a large degree of flexibility, efficiency, reliability, and cost effectiveness that enables extended and customized susceptibility testing, especially when facing the emergence of new resistance mechanisms. This is also supported by the poor performance of automated liquid-based systems to detect some carbapenemases (e.g., OXA-48-like and OXA-181-like carbapenemases) and the heteroresistance profiles. The advent of full automation in the clinical microbiology laboratory has revolutionized the process. In essence, several incentives have driven the advent of full automation, including needs for: (i) increased processing capacity; (ii) standardization of the process, which enables better costs control; (iii) optimized traceability; (iv) improved workflows; and (v) reduced turnaround times. Different reports have shown that most of these expectations have been achieved (14–18). Despite the increasing interest in disk diffusion AST, the lack of automation has prevented many microbiology laboratories from using this method. Now, a fully integrated and automated system for AST by disk diffusion is available. It allows (i) preparing an inoculum suspension by the Colibri; (ii) inoculating this suspension over the entire surface of Mueller-Hinton agar plates by the WASP; (iii) automatically dispensing antibiotic disks from a selection of up to 50 antibiotic disks loaded on the Radian in-line carousel; (iv) automatically incubating the culture plates on the WASPLab; (v) imaging the culture plates at specified time points; and (vi) extracting and interpreting inhibition zones diameters by the Radian Expert System based on EUCAST or CLSI breakpoints. In order to evaluate the accuracy of the fully automated disk diffusion AST provided by Copan, it was important to examine a representative number of clinical strains that are resistant to various antibiotic molecules, to assess the ability of this method to detect various resistance mechanisms, and to define the rate of major errors by assessing a significant number of susceptible strains. The overall categorical agreement between the Vitek 2 system and the Colibri coupled to Radian for the 718 nonduplicate clinical strains analyzed in the present study reached 99.1% (8,548/8,752; 95% CI 98.9% to 99.3%). The most important cause of very major errors encountered on the Vitek 2 for P. aeruginosa was related to the presence of heteroresistant populations. This finding has been previously highlighted in various reports (10, 12, 19). Heteroresistance constitutes a relevant cause of treatment failure, especially in recurrent or chronic infections. Thus, the risk of treatment failure is increased if the presence of a highly resistant subpopulation is not considered when prescribing antibiotics (10, 20–22). The rising global incidence of CPE constitutes a compelling challenge to public health because they are causing worse clinical outcomes. OXA-48-like carbapenemases belong to the Ambler class D β-lactamases. OXA-48-like carbapenemase has been reported in different *Enterobacterales* species and its transmission between species via plasmids is now clearly established (23). The OXA-48-like family is widely disseminated and constitutes a significant proportion of reported carbapenemases in different countries. However, OXA-48-like producers may be missed by some routine AST methods, due in part to their relative susceptibility to carbapenems and cephalosporins (23). Among the 43 CPE included in this study, one OXA-48-like, and one OXA-181-like were missed by the Vitek 2, even by rigorously applying the CPE screening cutoff defined by EUCAST. Other hallmarks of the phenotypic AST methods compared in the present study are summarized in Table 3. Another issue was encountered with cotrimoxazole and coagulase-negative staphylococci on the Vitek 2. This is a matter of concern that implies thorough and precautionary analysis. The Radian is not equipped with a cooling device, which highlights the importance of running internal quality controls using ATCC strains to assess the stability of the antibiotic disks. Our analysis of the internal quality controls using ATCC strains showed that the antibiotic disks were stable for at least 11 h 30 min after loading the Radian carousel, permitting a smooth management of the antibiotic cartridges. Finally, the fully automated solution for antimicrobial disk diffusion susceptibility enables respect for the EUCAST rule of 15-15-15 for the AST procedure.

**TABLE 3:** Hallmarks of the phenotypic AST methods compared in this study

Colibri coupled to Radian	VITEK 2 system
Fully automated method	Semiautomated method
Easy to change the antibiotics tested	
Greatest flexibility and cost-effectiveness	Less flexible and more expensive (susceptibility cards)
Reliable for detecting heteroresistant subpopulations	Low sensitivity for the detection of heteroresistant subpopulations
Easy to see test failures (e.g., mixed inoculum)	Purity check plates are mandatory (more consumable and additional workload)
More accurate detection of new resistance mechanisms	Problems in detecting some patterns of carbapenemases (e.g., OXA-48-like producers)
Applicable to many fastidious organisms	The range of drug dilution is usually very narrow
Inability to provide precise data regarding the level of an organism's resistance or susceptibility	Provides a good approximation of the MIC

All testing results are reported as percent agreement at the categorical level between the Vitek 2 system and the Colibri coupled to Radian under real routine laboratory conditions. The use of the percent agreement statistic may have some limitations, when considering a small number of determinations.

In conclusion, it has been pointed out that the workload requirements for Kirby-Bauer disk diffusion hindered its routine implementation in many clinical microbiology laboratories. However, the fully automated solution for antimicrobial disk diffusion provided by Copan will address these constraints with an accuracy that is equal to or better than that of the Vitek 2 system. By implementing the automation process in a stepwise manner (IT development, validation of the performances, staff training, and then routine implementation) we have become able to process 80% of our routine AST panels using the Colibri coupled to the Radian within 2 months.

In addition, this fully automated solution will facilitate the implementation of the EUCAST rapid AST directly from positive blood-culture bottles. Further studies are now needed to validate the EUCAST rapid AST using the Colibri coupled to the Radian, and to investigate the real impact of this protocol on the early adjustments of the antimicrobial regimen. Finally, the emergence of new antimicrobial resistance mechanisms, including some that may be difficult to detect, like carbapenemase production, implies that the analytical performances of the diagnostic devices should be iteratively reassessed and regularly challenged with internal and external quality controls to swiftly detect systematic errors.
