# Supplementary material for: Performance of Fully Automated Antimicrobial Disk Diffusion Susceptibility Testing Using Copan WASP Colibri Coupled to the Radian In-Line Carousel and Expert System
Source: J Clin Microbiol. 2021 Aug 18;59(9):e00777-21. doi: 10.1128/JCM.00777-21 (PMC8373016; doi:10.1128/JCM.00777-21)
Supplement: Supplemental file 1 — Fig. S1 to S5 and Table S1. Download JCM.00777-21-s0001.pdf, PDF file, 2.7 MB [file jcm.00777-21-s0001.pdf]

Performance of Fully Automated Antimicrobial Disk Diffusion Susceptibility Testing Using Copan WASP Colibri coupled to Radian in-Line Carousel and Expert System

Abdessalam CHERKAOUI[1], Gesuele RENZI[1], Nicolas VUILLEUMIER[2], and Jacques SCHRENZEL[1,3]

[1]Bacteriology Laboratory, Division of Laboratory Medicine, Department of Diagnostics, Geneva University Hospitals, 4 rue Gabrielle-Perret-Gentil, 1205 Geneva, Switzerland

[2]Division of Laboratory Medicine, Department of Diagnostics, Geneva University Hospitals and Faculty of Medicine, Geneva, Switzerland

[3]Genomic Research Laboratory, Division of Infectious Diseases, Department of Medicine, Geneva University Hospitals and Faculty of Medicine, Geneva, Switzerland

*__Corresponding author__: Abdessalam CHERKAOUI, PhD – FAMH, Bacteriology Laboratory, Division of Laboratory Medicine, Department of Diagnostics, Geneva University Hospitals, 4 rue Gabrielle-Perret-Gentil, 1205 Geneva, Switzerland

E-mail address: abdessalam.cherkaoui@hcuge.ch

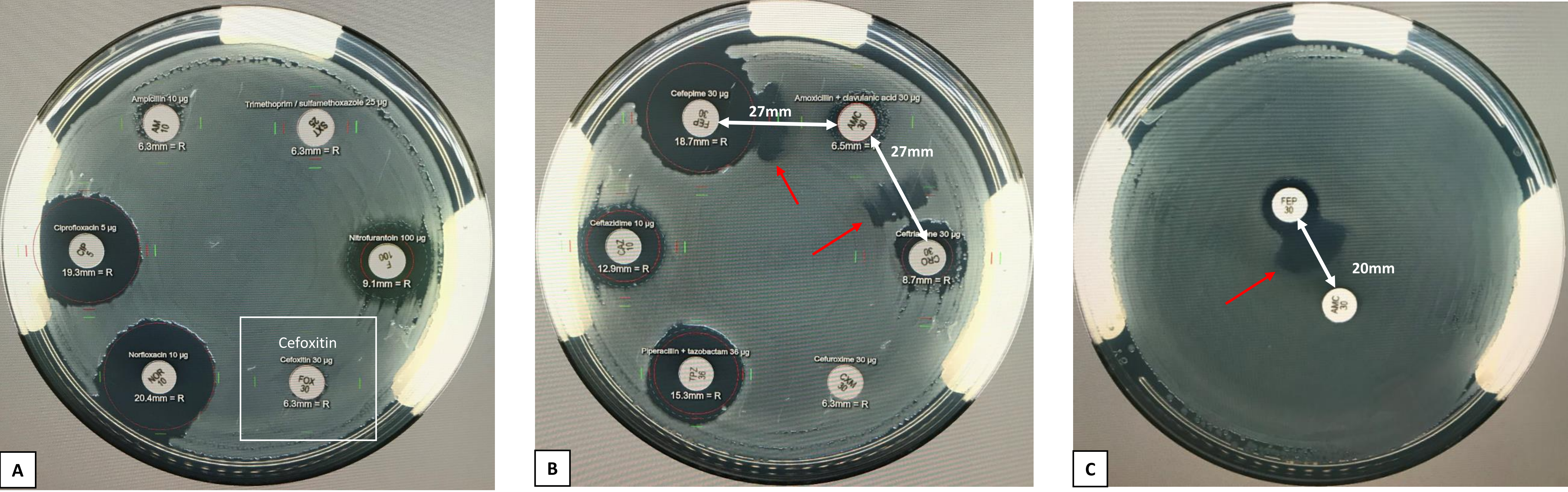

Supplementary material, Fig. S1: Double-disk synergy tests performed automatically by the Radian^{TM} and imaged by the WASPLab®.

(A) and (B) depict the primary MHE agar plates for *Enterobacter cloacae* complex strain; the amoxicillin-clavulanate disk (AMC) was automatically placed by the Radian^{TM} at 27 mm, center to center, of ceftriaxone (CRO) disk, and cefepime (FEP) disk. The inhibition zone around the CRO and FEP disks is enhanced (red arrows), highly suggesting the production of an ESBL.

(C) and (D) depict the DDST20: AMC disk was automatically placed by the Radian^{TM} at 20 mm, center to center, from FEP disk on MHE agar.
(C) The inhibition zone around the FEP disk is enhanced (red arrow), highly suggesting the production of an ESBL.
(D) The inhibition zone around the FEP disk is **NOT** enhanced (blue arrow), strongly arguing against the production of an ESBL.

ESBL confirmation methods used in this study
The DDST 27 mm is routinely performed on all strains.
The DDST 20 mm is carried out for the following conditions: 1) for all AmpC-producer strains, 2) for all strains isolated by rectal screening for ESBL and CPE, and 3) whenever the ESBL screening test is positive according to the EUCAST rules
We performed also the ESBL + AmpC Screen Kit 98008 (Rosco Diagnostica) to identify the partially de-repressed AmpC whenever the results of the DDST20 and cefoxitin were not conclusive.

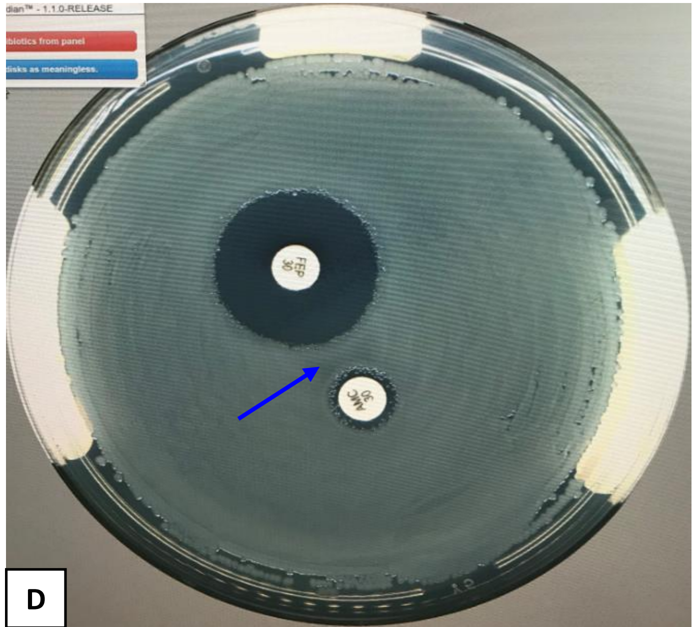

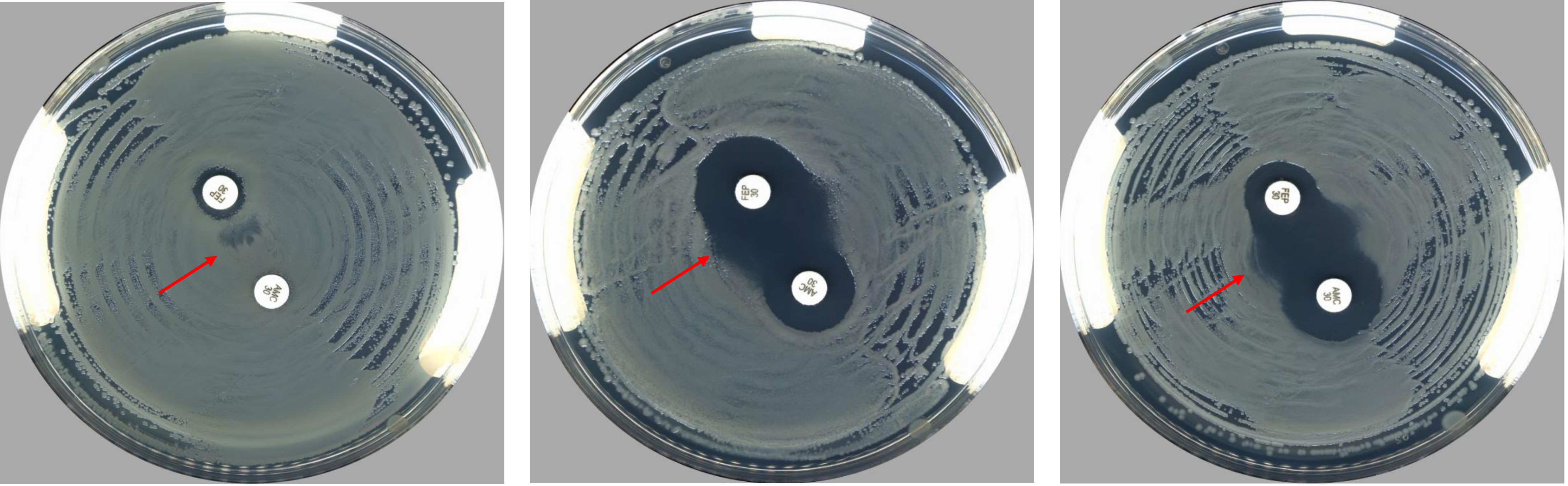

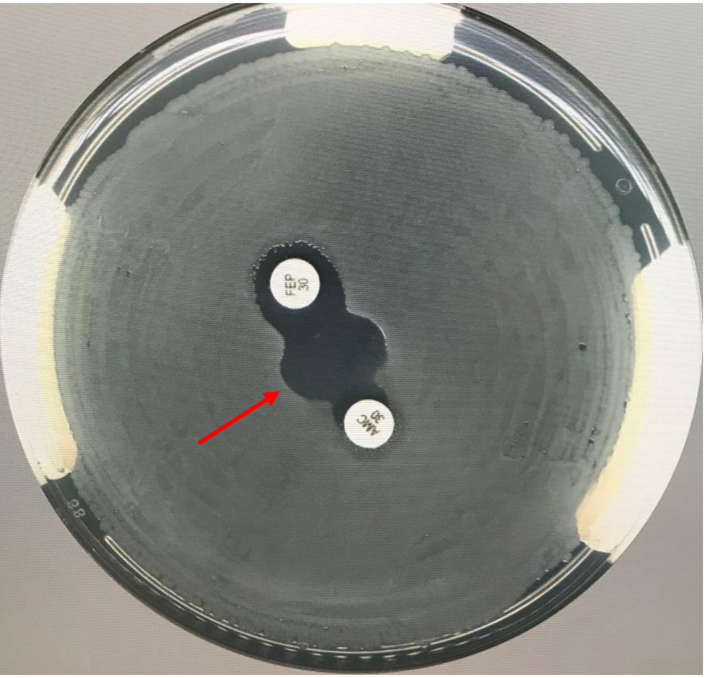

Supplementary material, Fig. S2: Other examples of positive DDST20. The inhibition zone around FEP disks is enhanced (red arrow), highly suggesting the production of ESBL

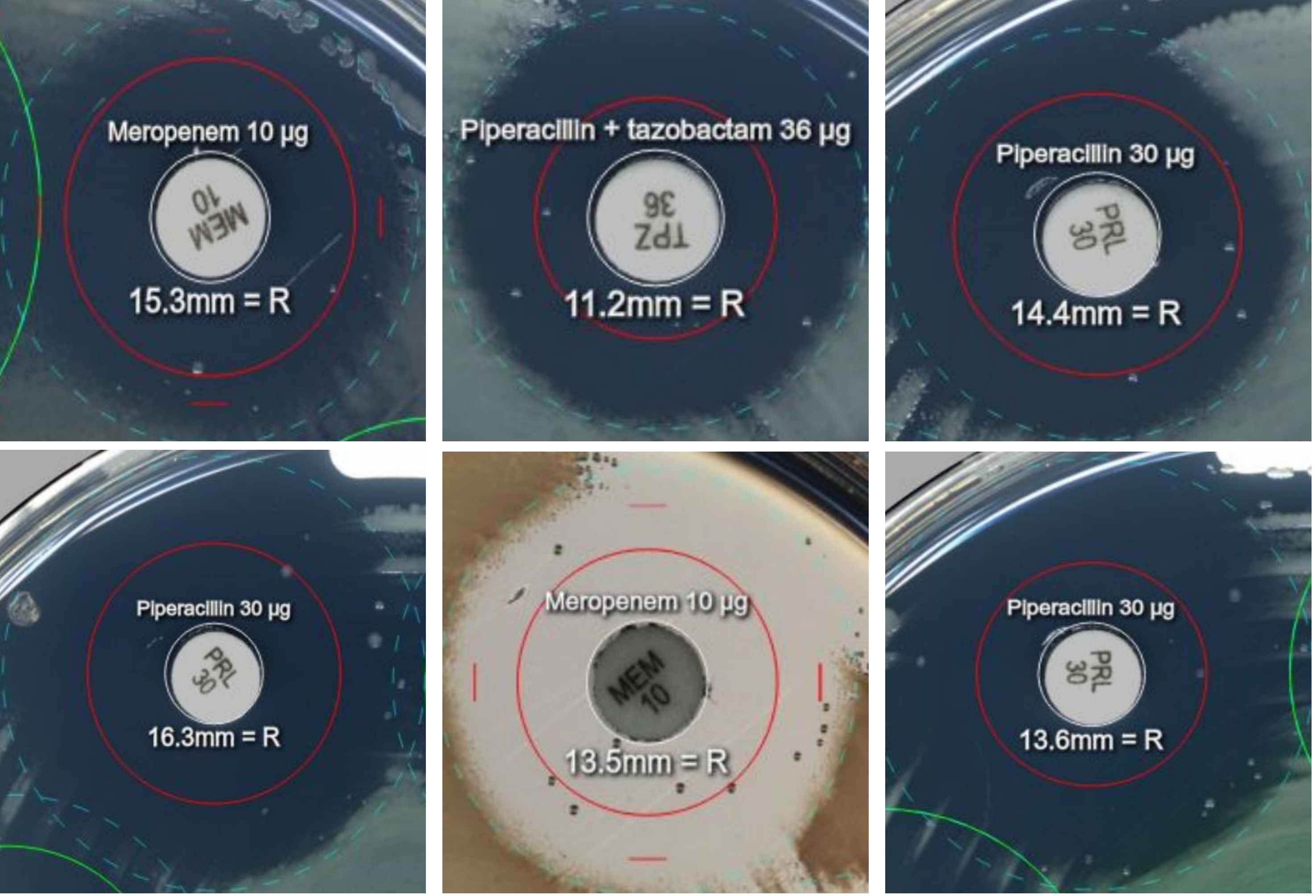

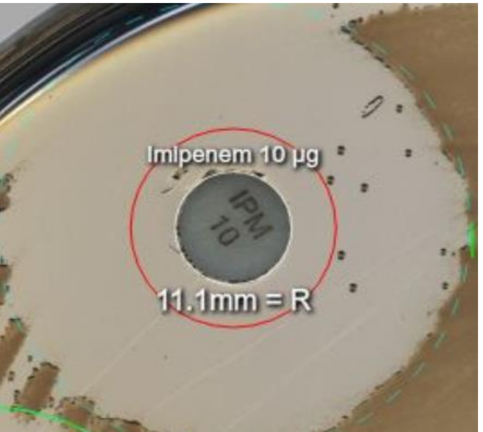

Supplementary material, Fig. S3: Examples of heteroresistant populations observed for several antibiotics in different *P. aerugonisa* strains included in this study.

The identification for all colonies visible within the inhibition halo was confirmed by MALDI-TOF MS to exclude any contamination.

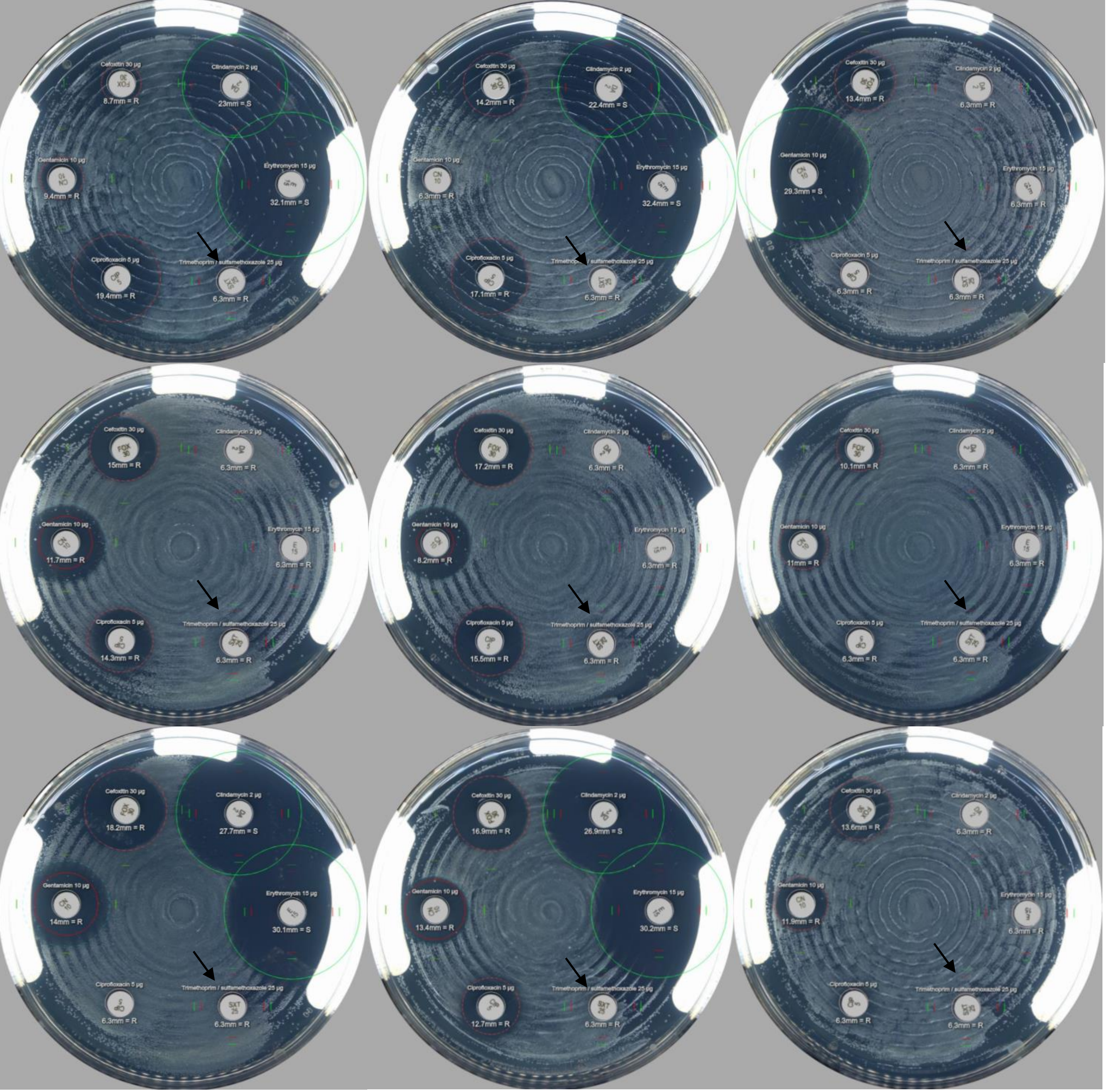

Species	VITEK® 2	Radian™		Thermo Scientific™ Sensititre™ MIC plate	
	Results	Inhibition zone diameter (mm)	Interpretation	MIC	Interpretation
Staphylococcus hominis	S	6	R	8	R
Staphylococcus epidermidis	S	6	R	12	R
Staphylococcus epidermidis	S	6	R	8	R
Staphylococcus epidermidis	S	6	R	8	R
Staphylococcus epidermidis	S	6	R	24	R
Staphylococcus epidermidis	S	6	R	8	R
Staphylococcus epidermidis	S	6	R	8	R
Staphylococcus epidermidis	S	6	R	8	R
Staphylococcus epidermidis	S	6	R	8	R
Staphylococcus epidermidis	S	10	R	8	R

R, resistant; S, susceptible

Supplementary material, Fig. S4: Discordant results for the co-trimoxazole (black arrow) between the compared methods

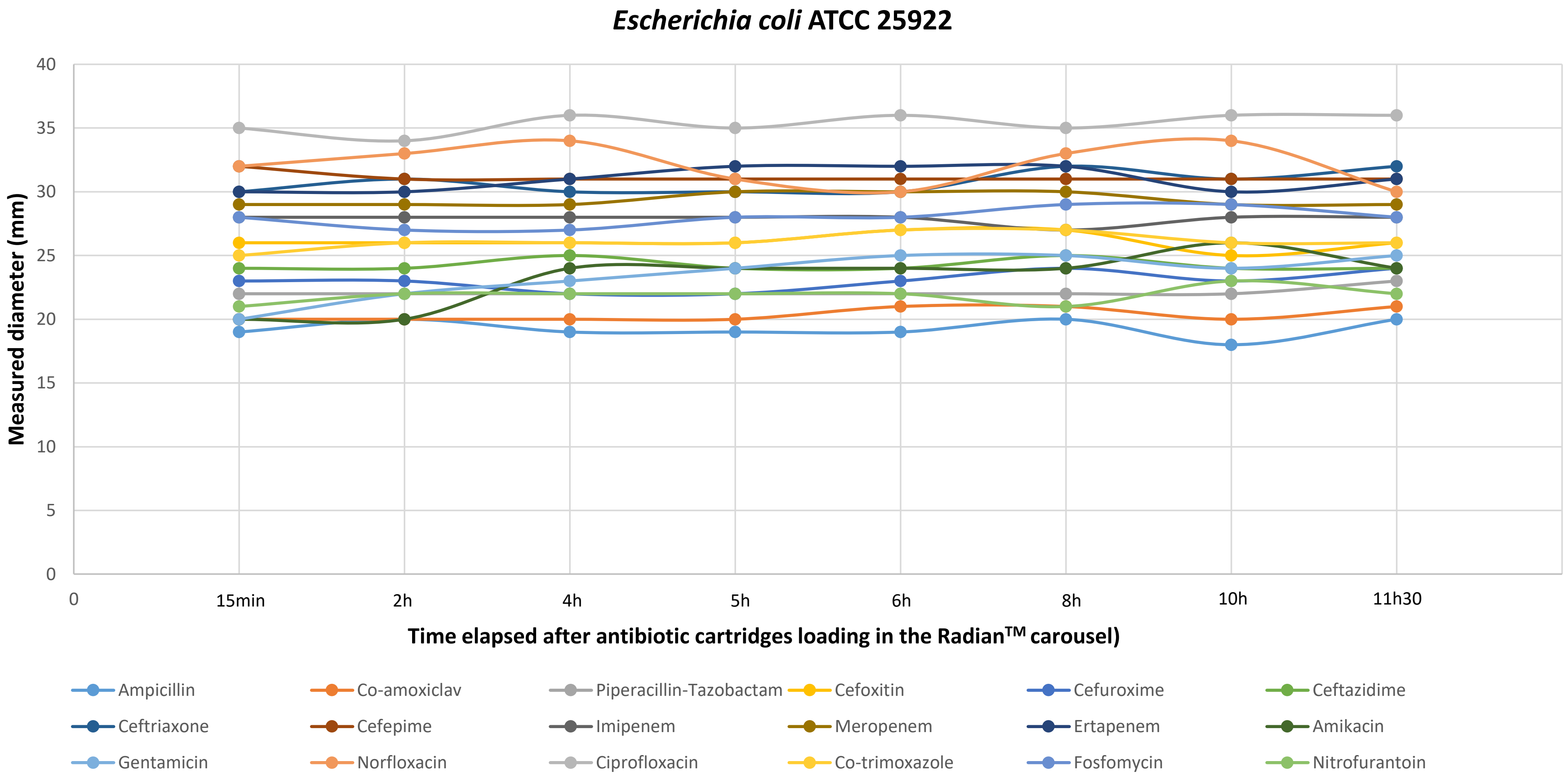

Supplementary material, Fig S5: Stability of antibiotics disks in the Radian™ carousel at specific time points corresponding to the time elapsed after antibiotic cartridges loading in the Radian™ carousel

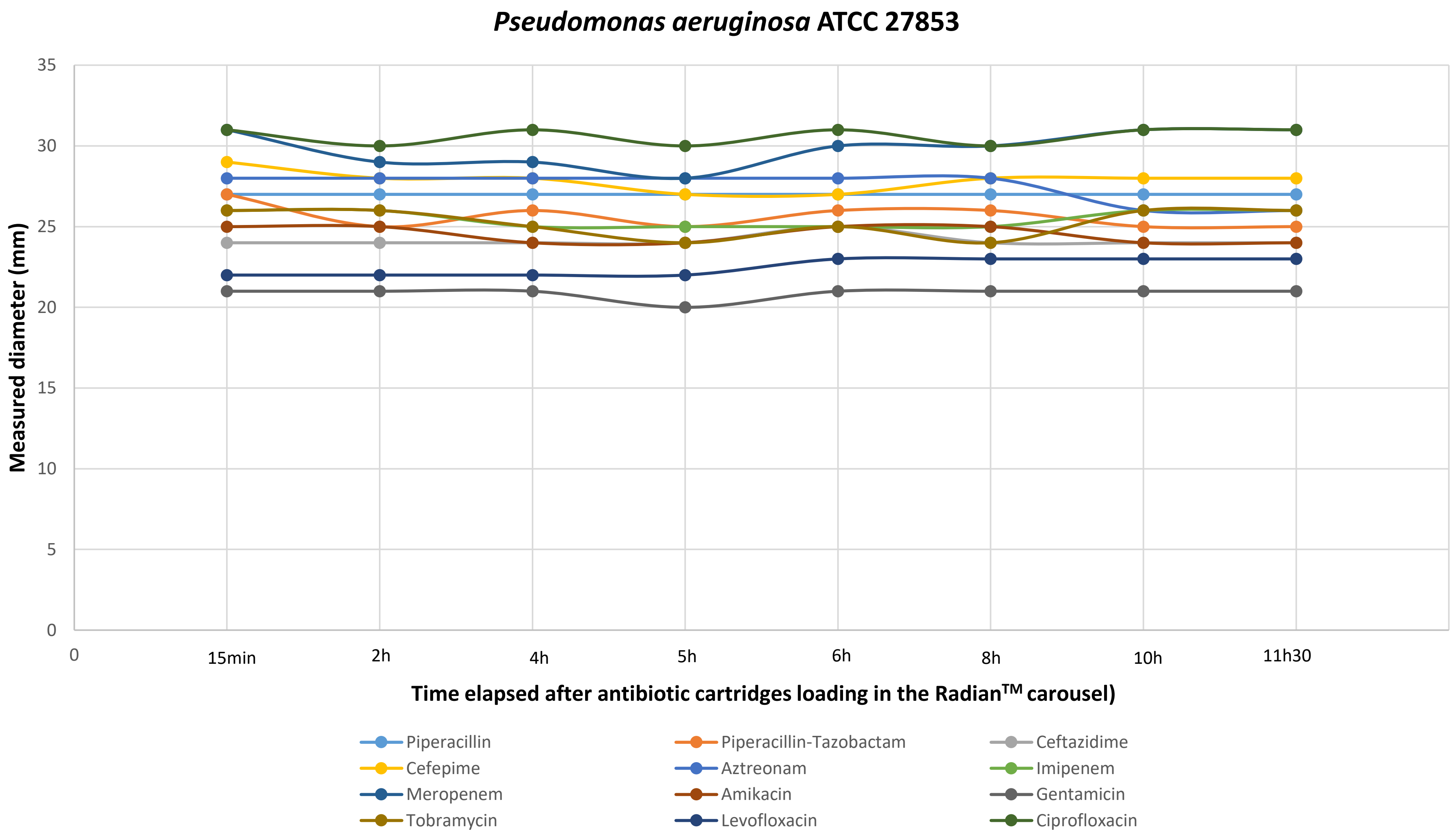

Pseudomonas aeruginosa ATCC 27853
Measured diameter (mm)
35
30
25
20
15
10
5
0
0
15min
2h
4h
5h
6h
8h
10h
11h30
Time elapsed after antibiotic cartridges loading in the RadianTM carousel)
Piperacillin
Piperacillin-Tazobactam
Ceftazidime
Cefepime
Aztreonam
Imipenem
Meropenem
Amikacin
Gentamicin
Tobramycin
Levofloxacin
Ciprofloxacin

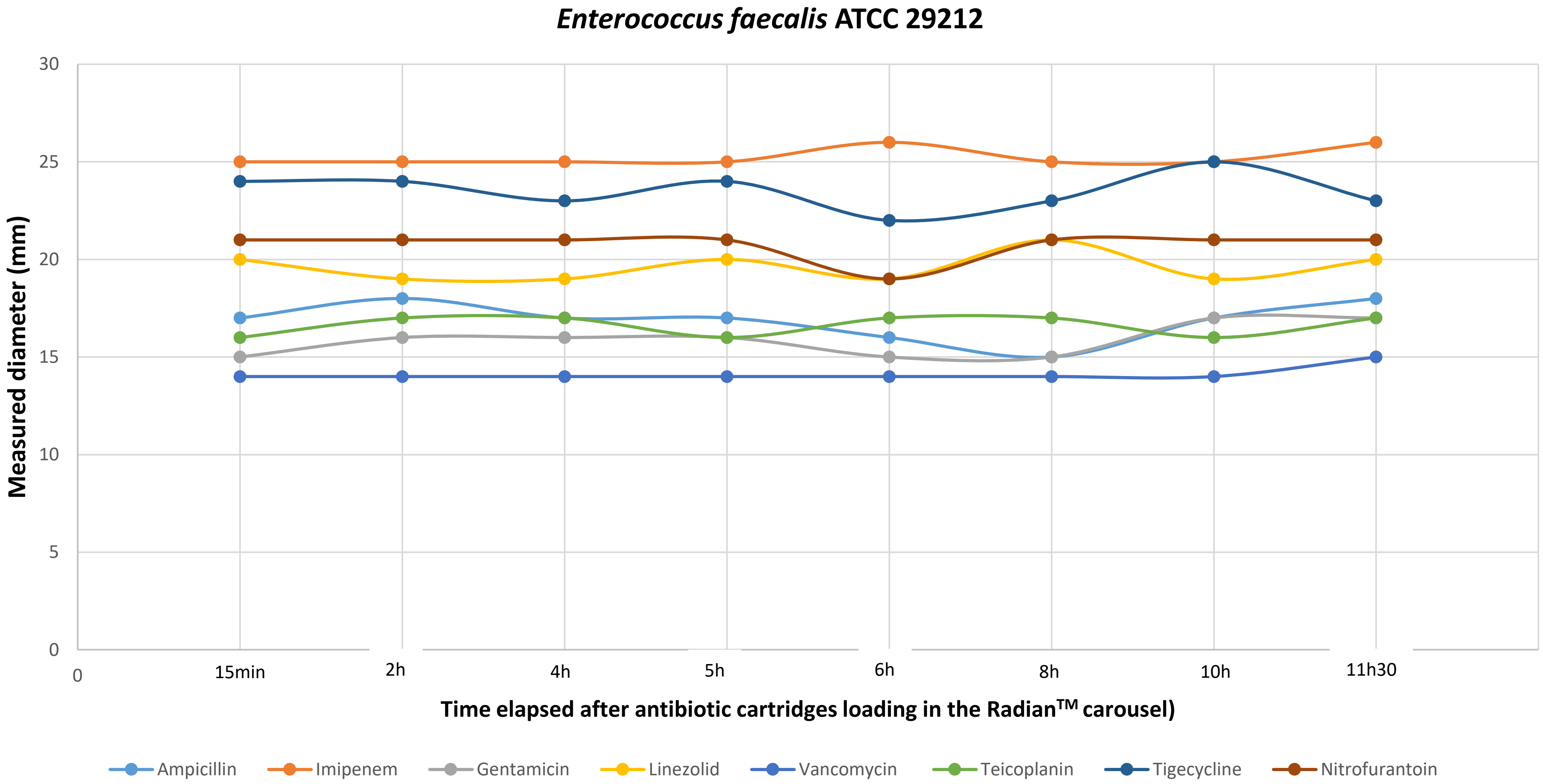

Enterococcus faecalis ATCC 29212
Measured diameter (mm)
30
25
20
15
10
5
0
0
15min
2h
4h
5h
6h
8h
10h
11h30
Time elapsed after antibiotic cartridges loading in the Radian™ carousel)
Ampicillin
Imipenem
Gentamicin
Linezolid
Vancomycin
Teicoplanin
Tigecycline
Nitrofurantoin

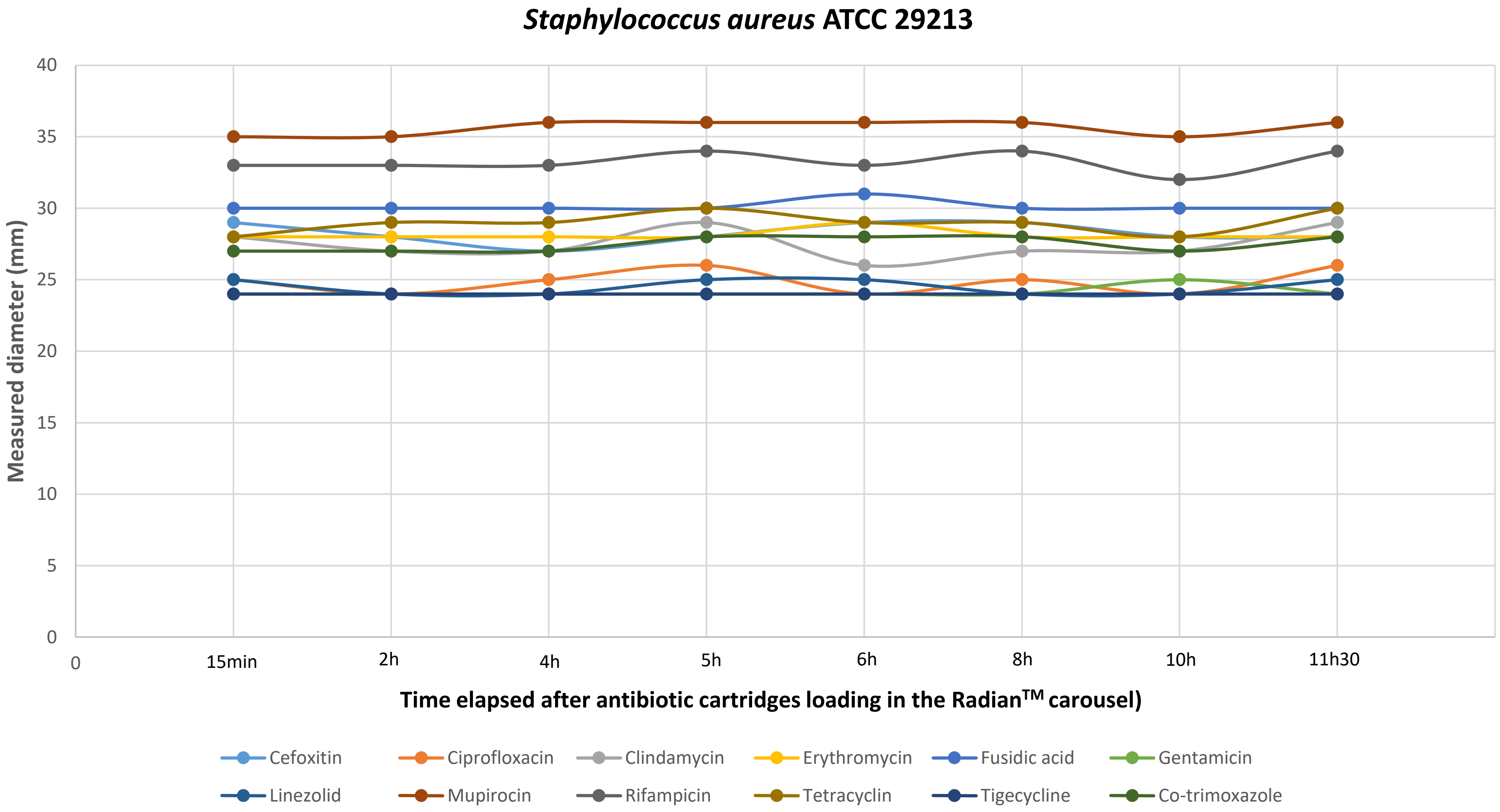

Staphylococcus aureus ATCC 29213
Measured diameter (mm)
40
35
30
25
20
15
10
5
0
0
15min
2h
4h
5h
6h
8h
10h
11h30
Time elapsed after antibiotic cartridges loading in the Radian™ carousel)
Cefoxitin
Ciprofloxacin
Clindamycin
Erythromycin
Fusidic acid
Gentamicin
Linezolid
Mupirocin
Rifampicin
Tetracyclin
Tigecycline
Co-trimoxazole

Escherichia coli producing OXA-181-like	Colibri™ coupled to Radian™		VITEK® 2	
**Antibiotic**	**Inhibition zone diameter (mm)**	**Interpretation**	**MIC**	**Interpretation**
Ampicillin	6	R	>=32	R
Amoxicillin/Clavulanate	6	R	>=32	R
Piperacillin/Tazobactam	9	R	>=128	R
Cefuroxime	18	R	16	R
Ceftazidime	23	S	<=1	S
Ceftriaxone	25	S	<=1	S
Cefepime	**23**	**R**	**<=1**	**S**
Imipenem	23	S	<=0.25	S
Meropenem	**23**	**S**	**<=0.25**	**S**
Ertapenem	**20**	**R**	**<=0.5**	**S**
Amikacin	20	S	<=2	S
Gentamicin	20	S	<=1	S
Norfloxacin	20	R	2	R
Ciprofloxacin	25	S	0.25	S
Co-trimoxazole	6	R	>=320	R

Escherichia coli producing OXA-48-like	Colibri™ coupled to Radian™		VITEK® 2	
**Antibiotic**	**Inhibition zone diameter (mm)**	**Interpretation**	**MIC**	**Interpretation**
Ampicillin	6	R	>=32	R
Amoxicillin/Clavulanate	6	R	>=32	R
Piperacillin/Tazobactam	12	R	64	R
Cefuroxime	18	R	8	R
Ceftazidime	25	S	<=1	S
Ceftriaxone	26	S	<=1	S
Cefepime	27	S	<=1	S
Imipenem	23	S	2	S
Meropenem	**24**	**S**	**<=0.25**	**S**
Ertapenem	**22**	**R**	**<=0.5**	**S**
Amikacin	23	S	<=2	S
Gentamicin	23	S	<=1	S
Norfloxacin	26	S	<=0.5	S
Ciprofloxacin	32	S	<=0.25	S
Co-trimoxazole	6	R	>=320	R

***Klebsiella pneumoniae* producing OXA-181-like**	**Colibri™ coupled to Radian™**		**VITEK® 2**	
**Antibiotic**	**Inhibition zone diameter (mm)**	**Interpretation**	**MIC**	**Interpretation**
Ampicillin	6	R	>=32	R
Amoxicillin/Clavulanate	6	R	>=32	R
Piperacillin/Tazobactam	6	R	>=128	R
Cefuroxime	20	S	4	S
Ceftazidime	25	S	<=1	S
Ceftriaxone	24	S	<=1	S
Cefepime	25	S	<=1	S
Imipenem	22	S	0.5	S
Meropenem	**22**	**S**	**1**	**S**
Ertapenem	**17**	**R**	**<=0.5**	**S**
Amikacin	20	S	<=2	S
Gentamicin	19	S	<=1	S
Norfloxacin	18	R	2	R
Ciprofloxacin	20	R	1	R
Co-trimoxazole	23	S	<=20	S

***Citrobacter koseri* producing OXA-181-like**	**Colibri™ coupled to Radian™**		**VITEK® 2**	
**Antibiotic**	**Inhibition zone diameter (mm)**	**Interpretation**	**MIC**	**Interpretation**
Ampicillin	6	R	>=32	R
Amoxicillin/Clavulanate	6	R	>=32	R
Piperacillin/Tazobactam	6	R	>=128	R
Cefuroxime	6	S	>=64	R
Ceftazidime	10	S	>=64	R
Ceftriaxone	6	S	>=64	R
Cefepime	12	S	32	R
Imipenem	24	S	2	S
Meropenem	**23**	**S**	**2**	**S**
Ertapenem	**19**	**R**	**<=0.5**	**S**
Amikacin	20	S	<=2	S
Gentamicin	20	S	<=1	S
Norfloxacin	6	R	>=16	R
Ciprofloxacin	8	R	>=4	R
Co-trimoxazole	6	R	>=320	R

Table-S1: Discordant results for carbapenemase-producing Enterobacteriaceae
